# Network Analysis Integrating microRNA Expression Profiling with MRI Biomarkers and Clinical Data for Prostate Cancer Early Detection: A Proof of Concept Study

**DOI:** 10.3390/biomedicines9101470

**Published:** 2021-10-14

**Authors:** Valeria Panebianco, Paola Paci, Martina Pecoraro, Federica Conte, Giorgia Carnicelli, Zein Mersini Besharat, Giuseppina Catanzaro, Elena Splendiani, Alessandro Sciarra, Lorenzo Farina, Carlo Catalano, Elisabetta Ferretti

**Affiliations:** 1 Department of Radiological Sciences, Oncology and Pathology, Sapienza University, Policlinico Umberto I, 00161 Rome, Italy; martina.pecoraro@uniroma1.it (M.P.); carnicelligiorgia@gmail.com (G.C.); carlo.catalano@uniroma1.it (C.C.); 2 Department of Computer, Control and Management Engineering, Sapienza University, 00161 Rome, Italy; paci@diag.uniroma1.it (P.P.); lorenzo.farina@uniroma1.it (L.F.); 3 Institute for Systems Analysis and Computer Science “Antonio Ruberti”, 00185 Rome, Italy; federica.conte@iasi.cnr.it; 4 Department of Experimental Medicine, Sapienza University, Policlinico Umberto I, 00161 Rome, Italy; zeinmersini.besharat@uniroma1.it (Z.M.B.); giuseppina.catanzaro@uniroma1.it (G.C.); elisabetta.ferretti@uniroma1.it (E.F.); 5 Department of Molecular Medicine, Sapienza University, Policlinico Umberto I, 00161 Rome, Italy; elena.splendiani@uniroma1.it; 6 Department of Maternal-Infant and Urological Sciences, Sapienza University, Policlinico Umberto I, 00161 Rome, Italy; alessandro.sciarra@uniroma1.it

**Keywords:** prostate cancer, magnetic resonance imaging, MR directed biopsy, network analysis, microRNA

## Abstract

The MRI of the prostate is the gold standard for the detection of clinically significant prostate cancer (csPCa). Nonetheless, MRI still misses around 11% of clinically significant disease. The aim was to comprehensively integrate tissue and circulating microRNA profiling, MRI biomarkers and clinical data to implement PCa early detection. In this prospective cohort study, 76 biopsy naïve patients underwent MRI and MRI directed biopsy. A sentinel sample of 15 patients was selected for a pilot molecular analysis. Weighted gene coexpression network analysis was applied to identify the microRNAs drivers of csPCa. MicroRNA–target gene interaction maps were constructed, and enrichment analysis performed. The ANOVA on ranks test and ROC analysis were performed for statistics. Disease status was associated with the underexpression of the miRNA profiled; a correlation was found with ADC (r = −0.51, *p* = 0.02) and normalized ADC values (r = −0.64, *p* = 0.002). The overexpression of miRNAs from plasma was associated with csPCa (r = 0.72; *p* = 0.02), and with PI-RADS assessment score (r = 0.73; *p* = 0.02); a linear correlation was found with biomarkers of diffusion and perfusion. Among the 800 profiled microRNA, eleven were identified as correlating with PCa, among which miR-548a-3p, miR-138-5p and miR-520d-3p were confirmed using the RT-qPCR approach on an additional cohort of ten subjects. ROC analysis showed an accuracy of >90%. Provided an additional validation set of the identified miRNAs on a larger cohort, we propose a diagnostic paradigm shift that sees molecular data and MRI biomarkers as the prebiopsy triage of patients at risk for PCa. This approach will allow for accurate patient allocation to biopsy, and for stratification into risk group categories, reducing overdiagnosis and overtreatment.

## 1. Introduction

Prostate cancer (PCa) is the fourth most common malignancy worldwide and the second cause of cancer related death in men [1]. Whole genome sequencing of over 1000 different prostate cancers in the past two decades yielded the identification of 97 significant gene mutations, in addition to seven hallmark signatures [2,3]. However, despite the extensive characterization, to date, more than 30% of prostate cancers remains unclassifiable [4,5].

Clinically significant PCa (csPCa) is often aggressive and potentially metastatic, requiring early detection and possibly multimodal therapy. Clinically insignificant PCa (ciPCa) often never progress and can be safely treated with active surveillance strategies.

Magnetic resonance imaging (MRI) is now recommended by the European Association of Urology (EAU) guidelines as a triage test to identify men with csPCa, as it has shown excellent results in terms of negative predictive value (NPV) and accuracy [6,7,8,9,10,11]. The MRI pathway has been largely validated in the PRECISION trial, the PROMIS trial, the MRI First and the 4M validating pairing studies [12,13,14,15]. A recent meta-analysis showed that MR directed biopsies (MRDB) detect a lower proportion of men with ciPCa [16], and is associated with a reduction by roughly 30–59% in unnecessary biopsies [6]. From evidence collected in the 2019 Cochrane meta-analysis, the MRI pathway proved to detect 12–20% more csPCa than a systematic biopsy. Nonetheless, MRI still misses around 11% of csPCa [7]. Guidelines also suggest the use of complementary noninvasive tests, namely, nomograms and blood or urine based biomarkers, to improvethe accuracy of MRI [6]. In this context, the addition of biomarkers in the MRI pathway could increase its NPV by up to >90%, avoiding unnecessary procedures [17]. MicroRNA (miRNA) constitute promising biomarkers for the early detection of PCa and for patients’ stratification, given their stability in tissue and biological fluids [18].

The necessity to integrate molecular data on PCa with imaging features can be accomplished using network analysis [19]. Weighted gene coexpression network analysis (WGCNA) is one of the most employed algorithms to analyze gene coexpression networks across gene expression data, exploring the association between network communities and phenotypic/clinical traits of interest [20,21]. MRI and omics analysis are emerging techniques in the modern panorama and constitute the state of the art technique for characterizing the outstanding heterogeneity of PCa, and design patient personalized strategies.

In the present cohort study, we comprehensively profiled tissue and circulating miRNA imaging biomarkers using MRI and clinical data, introducing a noninvasive diagnostic algorithm for the early diagnosis of csPCa.

## 2. Materials and Methods

### 2.1. Study Design

This was a prospective, single center cohort study with an observational analytical design. The Institutional Review Board and Ethical Committee approved the study, as being in line with the Declaration of Helsinki ethical principles. All patients were fully informed and signed informed consent prior to participation in the study. A total of 76 biopsy naïve patients, aged 47–82 years, with the suspicion of PCa were enrolled from July 2020 to January 2021. Within the entire cohort, we selected a sentinel sample of 15 patients in whom a first pilot molecular analysis was performed. Given the small size of the sample, careful patient selection was performed, to maximize results reproducibility. An equally sized cohort was selected: five patients with csPCa (Gleason Grade [GG] 7), five patients with ciPCa (GG 6), five patients with noncancerous pathology, and four healthy donors (HD, age range 55–62). Inclusion criteria were a total PSA (tPSA) elevation (≥3 ng/mL) and/or a positive digital rectal examination. Exclusion criteria were prior history of any metastatic disease and unfitness to perform MRI or MRDB. All selected patients underwent MRI and MRDB. Blood samples, and an additional tissue sample during the biopsy procedure, were collected for each patient. MicroRNA expression profiling was performed on total plasma (TP) extracellular vesicles (EV) and prostate tissue (T). For HD, only microRNA expression profiling data from EV and TP were available.

### 2.2. Imaging and Targeted Biopsy Protocols

All exams were performed on a 3-T MRI (GE Healthcare, Discovery 750, Milwaukee, WI, USA), using a multichannel surface phased array body coil (GE Healthcare, TORSOPA, Milwaukee, WI, USA). The multiparametric MRI protocol includes the acquisition of morphologic T2 weighted images (T2WI), functional diffusion weighted imaging (DWI)/apparent diffusion coefficient (ADC) map and dynamic contrast enhanced (DCE) sequences. Imaging acquisition protocol and image analysis were carried out according to the standards set in prostate imaging reporting and data system (PI-RADS) v2.1 [22]. Image analysis was carried out by two urogenital radiologists with five and ten years of experience in the field.

MRI quantitative parameters were collected for correlation with csPCa. Median ADC and normalized ADC (nADC) values were acquired from region of interest (ROI) traced on the ADC map. All the available sequences were used to accurately locate the lesion. In order to select an optimal reference for ADC normalisation, the radiologist used a previously validated methodology [23]. DCE quantitative pharmacokinetic parameters: Ktrans (influx transfer constant), Kep (efflux rate constant) and VE (fractional volume of extravascular extracellular space) were obtained using a dedicated software (OleaSphere), by placing ROIs traced on perfusion scans after selecting an appropriate arterial input function (AIF). Patients were submitted to MRI transrectal ultrasound (TRUS) targeted biopsy when a PI-RADS score ≥3 was reported. Out of those with lesions assigned a PI-RADS score equal to 3 (as the highest), only those with a PSA density (PSAD) ≥ 0.15 were referred to biopsy, after consultation with their referral urologist. Patient preparation for biopsy was conducted according to EAU guidelines [6]. A dedicated software (Trinity, Urostation Koelis, Grenoble, France) was used for navigation. The additional biopsy samples for the molecular analysis were stored in dry ice right after the collection. Histological analysis was performed by a pathologist with 15 years of experience in prostate cancer pathology.

### 2.3. Sample Collection and miRNA Profiling

Blood samples were obtained and stored in Ethylenediaminetetraacetic acid (EDTA) treated tubes, for both patients and controls. Samples were centrifuged to obtain plasma which was aliquoted and stored at −80 °C until further use. Samples of prostate tissue, obtained from biopsy patients only, and fresh frozen samples were processed for molecular analysis. RNA for microRNA evaluation was extracted from 500 μL of plasma for each sample. In detail, both circulating extracellular vesicles (EV) and total plasma (TP) were used for microRNA profiling.

EV were isolated from 500 μL of plasma using ExoQuick Plasma prep and Exosome Precipitation kit (#EXOQ5TM-1, System Biosciences, Palo Alto, CA, USA), following the manufacturer′s instructions.

RNA from TP and EV was extracted using plasma/serum RNA purification mini kit (#55000, Norgen, Thorold, ON, Canada). RNA was also obtained from biopsy tissue sample and was extracted from fresh frozen tissue using the Total Purification Plus Kit (#48300, Norgen, Thorold, ON, Canada). MicroRNAs profiling was performed using Nanostring nCounter Human v3 miRNA expression assay (NanoString Technologies, Seattle, WA, USA). The analysis of raw miRNA data was performed using nSolver 4.0 Software (NanoString, Seattle, WA, USA). Before data normalization, nCounter data quality control (QC) was assessed.

The mean + 1 standard deviation of negative controls was used for background thresholding, and normalization was performed by using the geometric mean of the five most stable microRNAs, identified through the RefFinder online tool [24] in TP and EV. For T data, instead, the normalization was performed using global normalization by the top 95 miRNAs, highly expressed (TOP100 function on nSolver).

### 2.4. Network Analysis

WGCNA R software package was used to analyze the correlation between differential microRNA expression and clinical and imaging traits of interest—age, BMI, tPSA, PSAD, PV, disease status, Gleason grade, max. dimension of the index lesion, extra-prostatic extension (EPE), PI-RADS score, ADC value, nADC value, ktrans, kep, Ve, neoplastic cell percentage, and overall mean cancer core length (MCCL). Soft thresholding was used to identify network communities; scale free topology criterion was applied [20,21]. 

For each gene, a “fuzzy” measure of module membership (MM) was defined by correlating its miRNA expression profile with the module eigengene (ME) of a given module. Membership of each gene to its ME is expressed on a scale from 0 to 1, the sign of the MM encodes the positive vs. the negative relationship with the module eigengene. To incorporate external information into the coexpression network, WGCNA makes use of gene significance (GS) measures computed as the correlations between miRNA expression and external sample traits. The higher the absolute value of GS on a scale from 0 to 1, the more biologically significant is that gene; the gene significance can take on positive or negative values.

### 2.5. miRNA–Target Interaction Network

The miRNA–target interaction networks were constructed and analyzed by exploiting MicroRNA ENrichment TURned NETwork (MIENTURNET, Rome, Italy) [25], a web tool designed to receive in input a list of miRNAs and infer possible evidences of their regulation on target genes based on both statistical and network based analyses. MIENTURNET produces a network that is computationally predicted and/or experimentally validated from TargetScan and miRTarBase, respectively.

### 2.6. Functional Enrichment Analysis

The functional enrichment analysis was performed by querying the Kyoto Encyclopedia of Genes and Genomes (KEGG) [26] pathway through MIENTURNET web tool [25]. P-values were adjusted with the Benjamini–Hochberg method and a threshold equal to 0.05 was set to identify functional annotations significantly enriched amongst genes of the input list.

### 2.7. RT-QPCR to Validate Differentially Expressed (DE) MicroRNAs 

To validate DE microRNAs, the RNA of 10 new samples was extracted from both EV and TP. Each microRNA was thus evaluated by reverse transcription quantitative polymerase chain reaction (RT-qPCR). Specifically, reverse transcription was initiated from 5 μL of extracted RNA using TaqMan™ MicroRNA Reverse Transcription Kit (Cat# 4366596, Applied Biosystem, ThermoFisher Scientific, Waltham, MA, USA) with a microRNA individual primer (Cat# 4427975 ID: 002284 *hsa-miR-138-5p*, Cat# 4427975 ID: 002743 *hsa-miR-520d-3p*, Cat# 4427975 ID: 001538 *hsa-miR-548a-3p*; ThermoFisher Scientific, Waltham, MA, USA). Subsequently, a mix composed of diluted cDNA template, ddPCR Supermix for Probes (no dUTP) (Cat# 1863025; Bio-Rad Laboratories) and TaqMan specific miRNA probe (Cat# 4427975, ThermoFisher Scientific, Waltham, MA, USA) was used to generate droplet by QX200TM Droplet Generator (Bio-Rad Laboratories, Hercules, CA, USA). PCR was carried out in a C1000 Touch Thermal Cycler (Bio-Rad Laboratories, Hercules, CA, USA) using the following thermal cycling program: 10 min at 95 °C for enzyme activation, followed by 45 cycles of 30 s at 94 °C and 1 min annealing/extension step at the appropriate temperature based on the primer/probe set (58 °C for *hsa-miR-548a-3p* and 56 °C for *has-miR-520d-3p* and *hsa-miR-138-5p*), 10 min at 98 °C for enzyme deactivation followed by infinite hold at 4 °C. Finally, the QX200 Droplet Reader (Bio-Rad Laboratories, Hercules, CA, USA) was used to read the fluorescence signals and QuantaSoft software was used for ddPCR data analysis (version 1.7.4; Bio-Rad Laboratories, Hercules, CA, USA).

### 2.8. Statistical Analysis

The nonparametric method one-way ANOVA on ranks (or Kruskal–Wallis test by rank) was used for comparing two or more samples of equal or different sample sizes. The diagnostic power of each miRNA was evaluated in terms of the receiver operating characteristic (ROC) probability curve analysis. The expression values of each predicted miRNA driver for the grading were logarithmically transformed and a “real association” was assigned according to their annotations: 1 for GG 6 patients, 0 for GG 7 patients. For a specified cut off (varying in the range of the miRNA expression values), the TPR and the FPR were computed, setting GG 7 as threshold value for csPCa. The ROC probability curves were drawn based on TPR and FPR measures at different cut offs, and the corresponding area under the curve (AUC) was computed. 

## 3. Results

The age range of the entire cohort ranged between 47 and 82, with an average age of 64.8 years. Mean PSA density (PSAD) was 0.17 ng/mL. The demographic and clinical characteristics of study participants are described in detail in Table 1.

Results on the entire cohort of patients, including five patients with noncancerous pathology, and four healthy donors are described in Supplementary File S1.

### 3.1. Analysis on the Cohort of Patients with PCa

#### 3.1.1. WGCNA on Extracellular Vesicles Data

**Figure 1:**
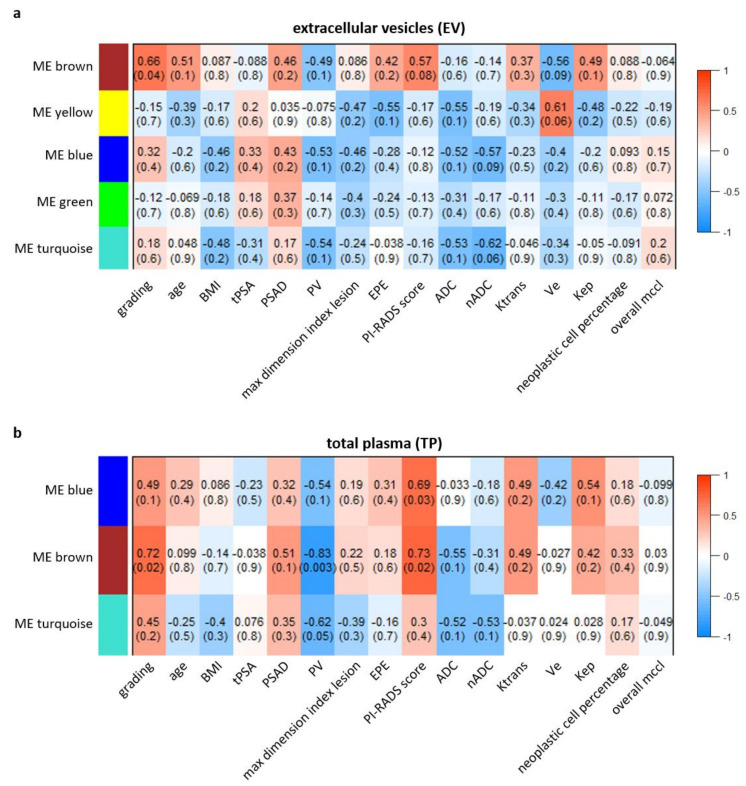
Weighted gene coexpression network analysis (WGCNA) on EV and TP data of PCa patients. Heatmap of the module–trait associations obtained by applying WGCNA on EV (**a**) and TP (**b**) data of PCa patients. In the heatmaps, each row corresponds to a module eigengene and each column to a trait of interest. Each cell contains the corresponding correlation and p-value. The heatmaps are color coded by correlation according to the color legend. EV, extracellular vesicles; TP, total plasma; PCa, prostate cancer; ME, module eigengene; BMI, body mass index; tPSA, total prostate specific antigen; PSAD, prostate specific antigen density; PV, prostate volume; EPE, extraprostatic extension; PI-RADS, prostate imaging reporting and data System; ADC, apparent diffusion coefficient; nADC, normalized apparent diffusion coefficient; MCCL, mean cancer core length.

**Figure 2:**
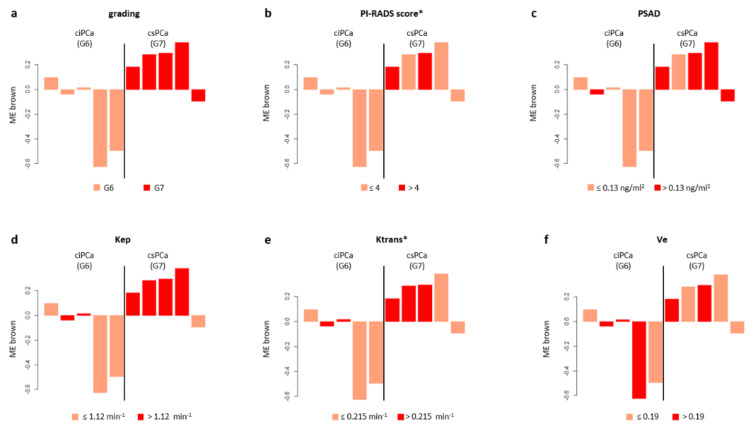
Module–trait association for EV data of PCa patients. Bar plots of the expression levels (*y*-axis) of brown ME across GG 6 and GG 7 PCa samples (*x*-axis). Expression levels of ME was log2 transformed and z-score normalized. Bar plots are coloured according with the different levels defined for grading (**a**), PI-RADS score (**b**), PSAD (**c**), Kep (**d**), Ktrans (**e**), Ve (**f**). For the color stratification of PSAD, Ve and Kep, we kept the median values used in the analysis of the whole cohort. For the color stratification of PI-RADS score, age and Ktrans, we used their medians across the GG 6-GG 7 PCa patients (asterisk). EV, extracellular vesicles; ME, module eigengene; PCa, prostate cancer; GG, Gleason grade; PSAD, prostate specific antigen density; PI-RADS, prostate imaging reporting and data system.

**Figure 3:**
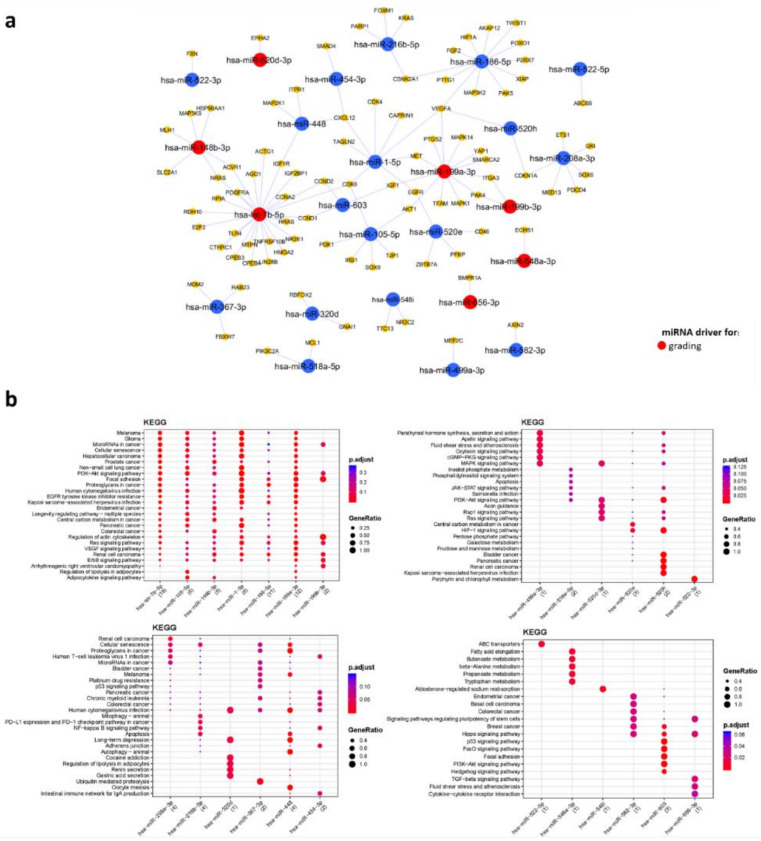
miRNA–target interaction network for miRNAs derived from EV data of PCa patients. (**a**) Network. The network shows the experimentally validated miRNA–target interactions retrieved from MIENTURNET. Blue dots represent miRNAs, yellow dots represent miRNA targets. Red color was used to highlight miRNAs found to be driver for grading. (**b**) KEGG pathways enrichment analysis. The main enrichment results for the targets of the miRNAs appearing in the network are presented as dot plots, where the *Y*-axis reports the annotation categories (i.e., KEGG pathways) and the *X*-axis reports the miRNAs with the number of recognized targets (i.e., number of targets with at least one annotation) in round brackets. The colors of the dots represent the adjusted p-values, whereas the size of the dots represents gene ratio (i.e., the number of miRNA targets found annotated in each category over the total number of recognized targets indicated in round brackets). EV, extracellular vesicles; PCa, prostate cancer; KEGG, Kyoto Encyclopaedia of Genes and Genomes.

The WGCNA analysis performed on circulating miRNAs derived from EV led to a coexpression network made of five modules ranging in size from 33 to 494 miRNAs. Displayed in Figure 1 are the module–trait correlations for the five modules identified. No correlation was found between differential microRNA expression and age (r = −0.048–0.51; *p* = 0.1–0.9). The brown module showed the highest statistical correlation with GG (r = 0.66; *p* = 0.04), indicating a significant association between high expression levels of miRNAs and csPCa (Figure 2a). However, the overexpression of miRNAs in this module was found to be positively correlated with PI-RADS (r = 0.57; *p* = 0.08), PSAD (r = 0.46; *p* = 0.2), Kep (r = 0.49; *p* = 0.1), Ktrans (r = 0.37; *p* = 0.3), and negatively correlated with Ve (r = −0.56; *p* = 0.09), despite not all values being statistically significant (Figure 1a and Figure 2b–g). To identify which miRNAs better represented the brown module, we selected those with MM > 0.7: we found that 58 out of 83 miRNAs constituting the brown module verified such a condition. A network of the experimentally validated miRNA–target interactions was constructed from the 58 selected miRNAs (Figure 3a). The functional enrichment analysis of the KEGG pathways in which their targets were involved was performed as the last step (Figure 3b). Note that the miRTarBase reported information on experimentally validated targets for only 25 out of 58 representative miRNAs. Among the most enriched pathways in which the targets of these 25 miRNAs were involved, we found “Chemokine signaling pathway”, “MicroRNAs in cancer”, “Prostate cancer” and several cancer related pathways, supporting their putative role as PCa specific miRNAs for subsequent use in noninvasive diagnostics. Importantly, the miRNA–target interaction network includes seven miRNA drivers for distinguishing between ciPCa (GG 6) and csPCa (GG 7) (Figure S1 and Figure 3a). These miRNAs were *hsa-let-7b-5p*, *hsa-miR-148b-3p*, *hsa-miR-656-3p*, *hsa-miR-199a-3p*, *hsa-miR-199b-3p*, *hsa-miR-548a-3p*, and *hsa-miR-520d-3p*.

#### 3.1.2. WGCNA on Total Plasma Data

**Figure 4:**
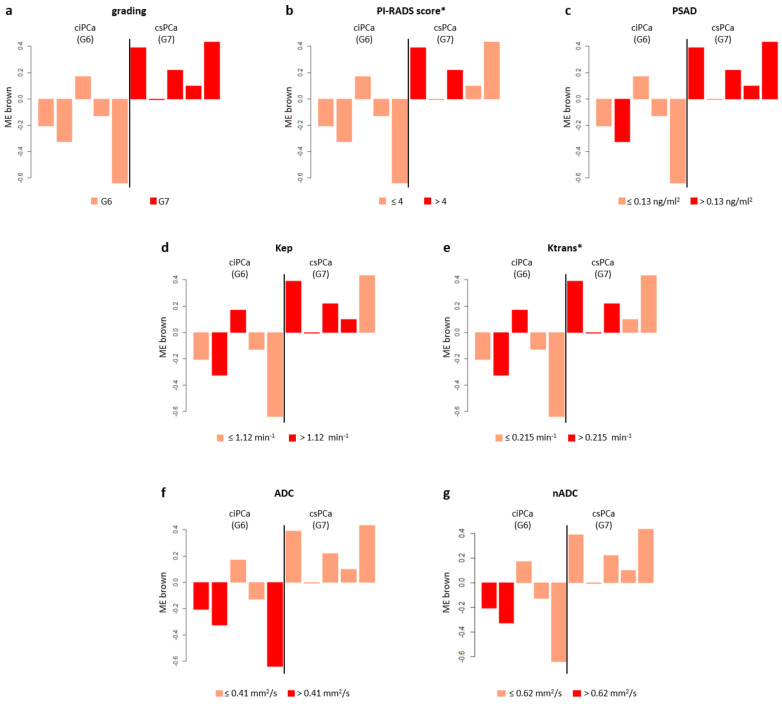
Module–trait association for TP data of PCa patients. Bar plots of the expression levels (*y*-axis) of brown ME across GG 6 and GG 7 PCa samples (*x*-axis). Expression levels of ME were log2 transformed and z-score normalized. -Bar plots are coloured according with the different levels defined for grading (**a**), PI-RADS score (**b**), PSAD (**c**), Kep (**d**), Ktrans (**e**), ADC (**f**), nADC (**g**). For the color stratification of PSAD, ADC, nADC and Kep, we kept the median values used in the analysis of the whole cohort. For the color stratification of PI-RADS score and Ktrans, we used their medians across the GG 6-GG 7 PCa patients (*). TP, total plasma; PCa, prostate cancer; ME, module eigengene; PSAD, prostate specific antigen density; PI-RADS, prostate imaging reporting and data system; GG, Gleason grade; ADC, apparent diffusion coefficient; nADC, normalized apparent diffusion coefficient.

The WGCNA analysis performed on circulating miRNAs derived from TP led to a coexpression network made of three modules ranging in size from 36 to 573 miRNAs (Figure 1b). No correlation was found between differential microRNA expression and age (r = −0.25–0.29; *p* = 0.4–0.5). We found that the brown module had the strongest overall statistical correlation with the Gleason grade (r = 0.72; *p* = 0.02), and that miRNAs overexpression in this module was predictive of csPCa (Figure 4a). A statistical significance was found for PI-RADS (r = 0.73; *p* = 0.02) and PSAD (r = 0.51; *p* = 0.1); other quantitative parameters of perfusion, Kep (r = 0.42; *p* = 0.2), and Ktrans (r = 0.49; *p* = 0.2) positively correlated with miRNA overexpression (Figure 1b and Figure 4b–e). A moderate negative correlation, instead, was observed between the ADC and nADC values and the brown ME (r = −0.55, *p* = 0.1; r = −0.31, *p* = 0.4, respectively), although statistical significance could not be achieved for these parameters (Figure 1b and Figure 4f–g). MicroRNAs representative of the brown module were identified by setting as threshold of significance MM > 0.7. Interestingly, 35 out of 36 miRNAs belonging to the brown module verified this condition, indicating that this module is topologically well defined and easily detectable, being composed by tightly interconnected nodes with intriguing patterns of molecular coabundance.

**Figure 5:**
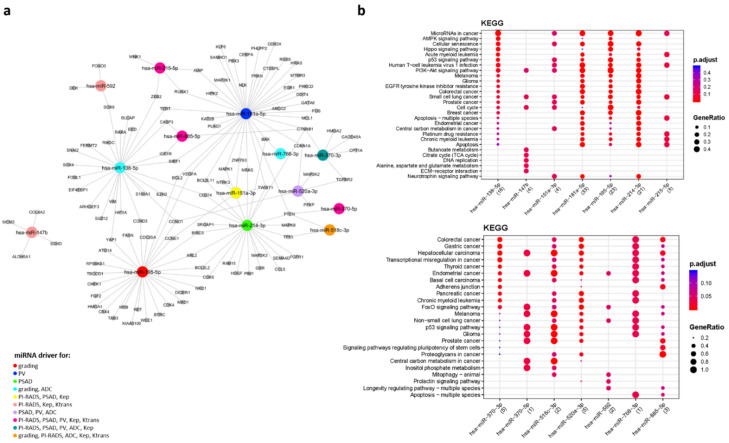
miRNA–target interaction network for miRNA derived from TP data of PCa patients. (**a**) Network. The network shows the experimentally validated miRNA–target interactions retrieved from MIENTURNET. Dots representing miRNAs are colored according with their associations with the external traits of interest. (**b**) KEGG pathways enrichment analysis. The main enrichment results for the targets of the miRNAs appearing in the network are presented as dot plots, where the *Y*-axis reports the annotation categories (i.e., KEGG pathways) and the *X*-axis reports the miRNAs with the number of recognized targets (i.e., number of targets with at least one annotation) in round brackets. The colors of the dots represent the adjusted p-values, whereas the size of the dots represents gene ratio (i.e., the number of miRNA targets found annotated in each category over the total number of recognized targets indicated in round brackets). TP, total plasma; PCa, prostate cancer; KEGG, Kyoto Encyclopaedia of Genes and Genomes; tPSA, total prostate specific antigen; PSAD, prostate specific antigen density; PV, prostate volume; PI-RADS, prostate imaging reporting and data system; ADC, apparent diffusion coefficient; nADC, normalized apparent diffusion coefficient.

The biological relevance of these miRNAs was assessed by constructing a miRNA target interaction network (Figure 5a) and performing functional enrichment analysis (Figure 5b). Note that miRTarBase reported information on experimentally validated targets only for 14 out of 35 representative miRNAs. Among the most enriched pathways in which the targets of these 14 miRNAs were “MicroRNAs in cancer”, “Prostate cancer” and various signaling pathways frequently altered in cancer, such as the *PI3K–Akt* signaling pathway, *p53* signaling pathway, and *FoxO* signaling pathway (Figure 5b). The miRNA–target interaction network includes four miRNA drivers for Gleason score, hence that could be used to distinguish between patients with csPCa and ciPCa (Figure 5a). These are *hsa-miR-518c-3p*, *hsa-miR-195b-5p*, *hsa-miR-138-5p*, and *has-miR-766-3p*. At the ROC analysis, these four miRNA drivers for grading reached an AUC > 0·91 (Figure S2).

### 3.2. DEMs Validation Using RT-QPCR

**Figure 6:**
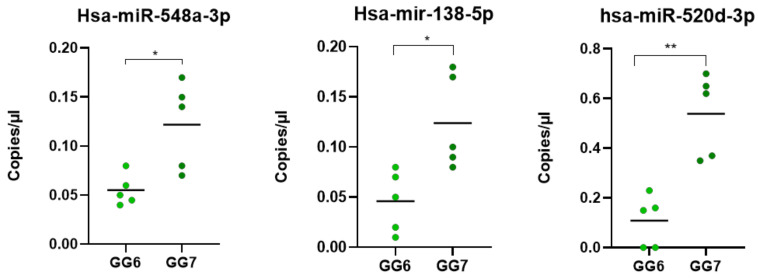
The set of miRNAs validated in as additional cohort of 10 patients affected by prostate cancer. GG, Gleason grade. * *p* < 0.01; ** *p* < 0.001.

Among the eleven initially identified microRNA, three were further validated in a new cohort of ten PCa patients (five csPCa and five ciPCa). *Hsa-miR-548a-3p*, *has-miR-138-5p* and *has-miR-520d-3p* were confirmed as significant (*p* < 0.5) circulating biomarkers of prostate cancer with higher values in the new series of G7 patients compared to G6 patients, using the droplet digital PCR, as shown in Figure 6.

## 4. Discussion

In this single center cohort prospective study, we applied miRNA profiling to MRI biomarkers and clinical data by using a network-based approach.

In the entire cohort, the MRI biomarkers that most significantly correlated with differential miRNA expression were the ADC and the nADC values. Values lower than the median nADC (0.62 × 10^−3^mm^2^/s) significantly correlated with a higher expression of driver miRNAs in the group of patients with disease status (PCa and non-PCa). As expected, the lowest values were found in PCa patients, suggesting that ADC could be the one biomarker distinguishing PCa from non-PCa lesions. This value is in line with previously published works, confirming the role of low ADC values for csPCa diagnosis [23,27,28]. The MiRNA drivers of disease status could distinguish healthy donors from patients with either ciPCa or csPCa.

The most noteworthy results were found when analyzing the PCa patient cohort: in this subgroup, the most representative data came from plasma miRNA expression profiling, highlighting the applicability of our methods to clinical practice.

The analysis of the global trends of the expression of the MEs of interest showed that a clear distinction can be made between csPCa and ciPCa: miRNAs in these modules were consistently upregulated for csPCa; instead, the trend was opposite in ciPCa.

Of note is that PI-RADS v2.1 category assignment to index lesions was consistent with these patterns of expression, reflecting the accuracy of the PI-RADS score for csPCa detection [22]. Global expression analysis also revealed that accurate predictors of csPCa were quantitative imaging features, notably, the perfusion parameters, indicative of increased vascularity and angiogenesis. Specifically, in our samples, the values of median Kep greater than 1.12 min^−1^ constituted a cut off value predictive of csPCa. Similarly, values of median Ktrans > 0.215 min^−1^ could consistently distinguish significant from insignificant cancer lesions. Our results are similar to what was found in other investigations. Among many, Hötker et al. [29] reported a median Ktrans >0.210 min^−1^ as a cut off value for csPCa diagnosis and Wei et al. [30] a Ktrans cut off value of 0.205 min^−1^ for PCa detection. As in the whole cohort, the analysis of differentially expressed miRNAs in the brown module from plasma showed that csPCa was also characterized by a significant reduction in the ADC values of the index lesions. In a recently published meta-analysis, Meyer et al. [28] showed that the pooled mean ADC value of the csPCa was 0.86 × 10^−3^ mm^2^/s (95% CI 0.83–0.90), while the pooled mean ADC value of ciPCa was 1.1 × 10^−3^ mm^2^/s [95% CI 1.03–1.18]. The low cut-off of our cohort is probably linked to the small size of the sample; however, it correctly implies that, for high grade tumors, the ADC value is low.

The clinical biomarker predictive of csPCa was the PSAD: we observed that levels higher than 0.13 ng/mL were associated with the probability of csPCa. PSAD is one of the strongest predictors of csPCa; in several risk models and studies, PSAD and the PI-RADS score were significant independent predictors of csPCa at biopsy [31,32,33]. The data described above were confirmed by the heatmap analysis. The selected modules of interest were only those showing a statistical association with the Gleason score and csPCa. In the heatmaps, regarding imaging features, we found that drivers of PCa status were also drivers of a higher PI-RADS score, with a significant strength of correlation. CsPCa also showed a linear relationship with higher Kep, Ve and Ktrans values, although we were not able to reach a statistical significance for these latter parameters. In addition, negative correlations of ADC and nADC values with csPCa were confirmed in all modules. The evidence collected in our study is in line with a previous landmark work from Stoyanova et al. [34] who reported the same patterns of association between imaging biomarkers and PCa molecular characteristics that we were able to highlight. Given the relation between csPCa molecular features and MRI biomarkers, quantitative imaging analysis could be of aid, especially, in the evaluation of equivocal PI-RADS 3 cases, or in subjects who are at higher risk. Indeed, the MRI features of aggressive PCa are positively associated with the level of expression of genetic markers of poor prognosis, as recently underlined by Norris et al. [35].

Out of the 800 miRNAs profiled, we identified eleven differentially expressed miRNA that correlated with grading, which could constitute sensitive biomarkers of csPCa. We looked for possible mechanisms of differential expression of the 11 miRNAs. First, we queried the GDC TCGA prostate cancer (PRAD) dataset through the Xena database for copy number alterations of each of these miRNAs. We also looked for alterations in the regulatory regions of the genes coding for these miRNAs on the Transcription Regulatory Region Database (TRRD). We did not find copy number alterations nor regulatory regions alterations for all of them. Therefore, we hypothesize that other regulatory mechanisms can be involved in their differential expression, such as different levels of promoter methylation and/or post-transcriptional mechanisms [36]. As displayed in the miRNA–target gene interaction maps, these differentially expressed MicroRNAs (DEmiRNAs) are also drivers of imaging biomarkers, further endorsing the value of mpMRI as a triage test. Within this group of selected DEMs, evidence in literature already exists on the possible role of *let-7b-5p* as biomarker specific of aggressive cancers [37,38], *miR-195-5p* [39,40], *miR-148b-3p* [38,41], *miR-518c-3p* [42,43] as biomarkers specific of csPCa.

The validation of the identified miRNAs, on a larger cohort, could lead to a paradigm shift in the diagnostics of PCa. The association of omics data, obtained from plasma circulating miRNAs (liquid biopsy), with MRI biomarkers could further enhance prostate cancer early detection by providing an accurate patient risk stratification. In detail, three risk groups (low, intermediate, and high risk) could be identified according to (i) the level of expression of the miRNAs recognized to correlate with csPCa, and (ii) MRI biomarkers positivity range (PI-RADS score, ADC value, and perfusion pharmacokinetic parameters).

Limitations of the study need to be acknowledged: a small sample size, single center study and small sample heterogeneity (GG > 7 were excluded due to the low prevalence in the general population, that, otherwise, would affect the reproducibility of the obtained data). The evaluation of the accuracy of the selected markers integrated in the MRI pathway will be the object of upcoming studies on a wider population sample and should ideally be followed by randomized controlled trials.

## 5. Conclusions

Gene–imaging interaction analysis could offer a valuable tool for PCa diagnosis. By integrating clinical data, MRI biomarkers and miRNA expression, through a network-based approach, we identified eleven differentially expressed miRNAs, which could represent sensitive biomarkers for csPCa early detection.

We propose a diagnostic paradigm shift that sees molecular data as adjunct to MRI biomarkers during the prebiopsy triage of patients at risk for PCa. The clinical implications of this approach would be a dramatic reduction in the number of unnecessary biopsies, and more accurate patient stratification. This integrative approach will ultimately reduce PCa associated overdiagnosis, overtreatment and overall related costs.

## Figures and Tables

**Table 1:** Demographic and clinic radiologic characteristics of the study participants. PSA, prostate specific antigen; SD, standard deviation; PCa, prostate cancer; PI-RADS, prostate imaging reporting and data system; TURP, transurethral resection of the prostate.

Variable	Biopsed Cohort
Mean age (years), SD	64.8 ± 9.40
Mean PSA value (ng/mL), SD	7.62 ± 2.59
Mean PSA density (ng/mL^2^), SD	0.17 ± 0.12
Mean prostate volume (ml), SD	57.6 ± 36.43
PCa-negative, *n* (%)	5 (33)
PCa-positive, *n* (%)	10 (67)
Gleason grade 1, *n* (%)	5 (33)
Gleason grade 2, *n* (%)	5 (33)
Peripheral zone, *n* (%)	13 (87)
Transition zone, *n* (%)	2 (13)
PI-RADS scoring:	
PI-RADS 3, *n* (%)	5 (33)
PI-RADS 4, *n* (%)	8 (54)
PI-RADS 5, *n* (%)	2 (13)
Prior TURP, *n*	2 (13)

## Data Availability

The data that support the findings of this study are available from the corresponding author, (VP), upon reasonable request.

## Supplementary Materials

The following are available online at https://www.mdpi.com/article/10.3390/biomedicines9101470/s1, Figure S1: ROCs analysis for microRNAs detected in the EV brown module, Figure S2: ROCs analysis for microRNAs detected in the TP brown module. File S1: Additional results on the entire cohort of patients, including five patients with non-cancerous pathology, and four healthy donors (HD, age range 55–62).
