# Supplementary material for: Network Analysis Integrating microRNA Expression Profiling with MRI Biomarkers and Clinical Data for Prostate Cancer Early Detection: A Proof of Concept Study
Source: Biomedicines. 2021 Oct 14;9(10):1470. doi: 10.3390/biomedicines9101470 (PMC8533640; doi:10.3390/biomedicines9101470)

hsa-let-7b-5p

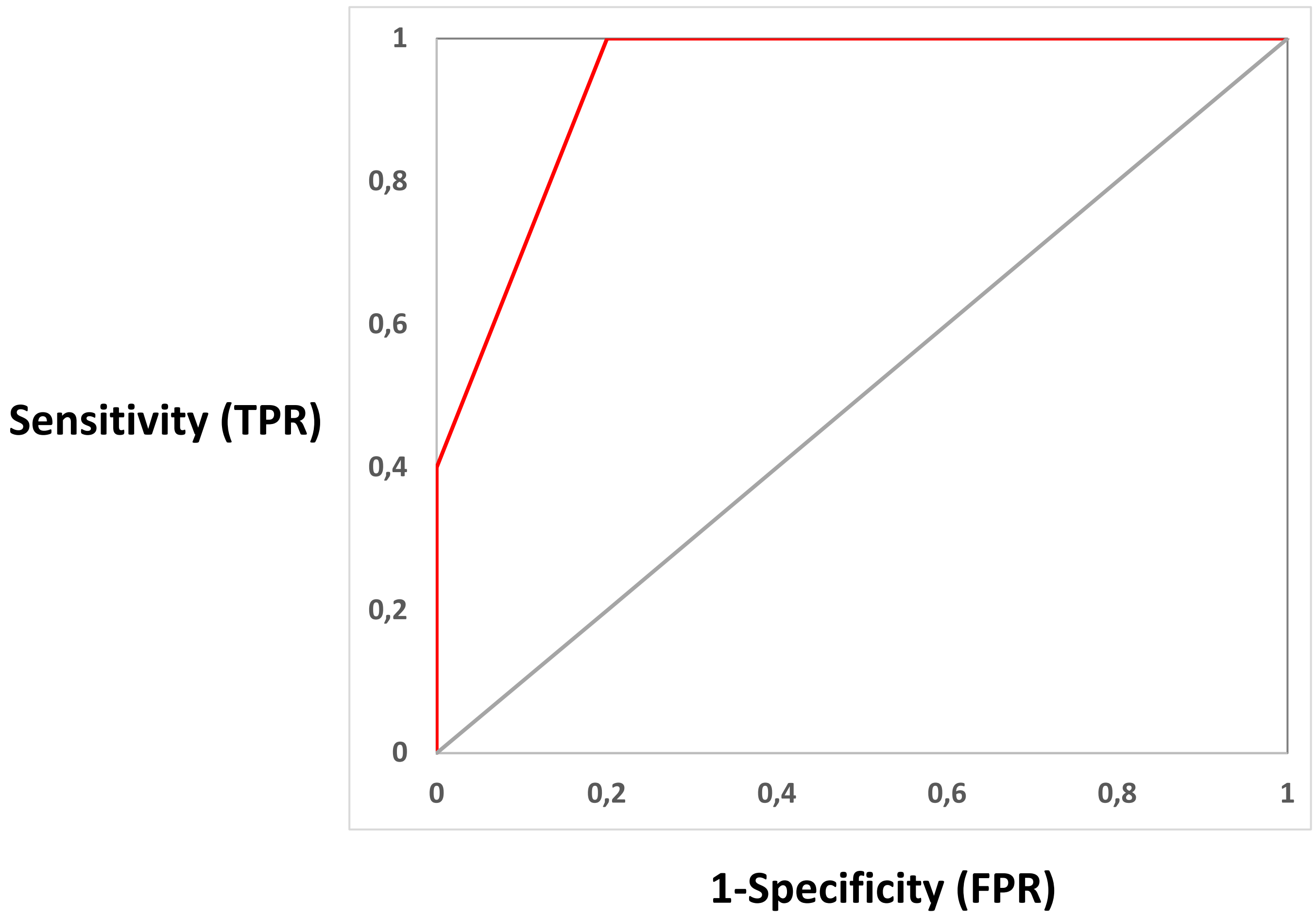

hsa-miR-148b-3p

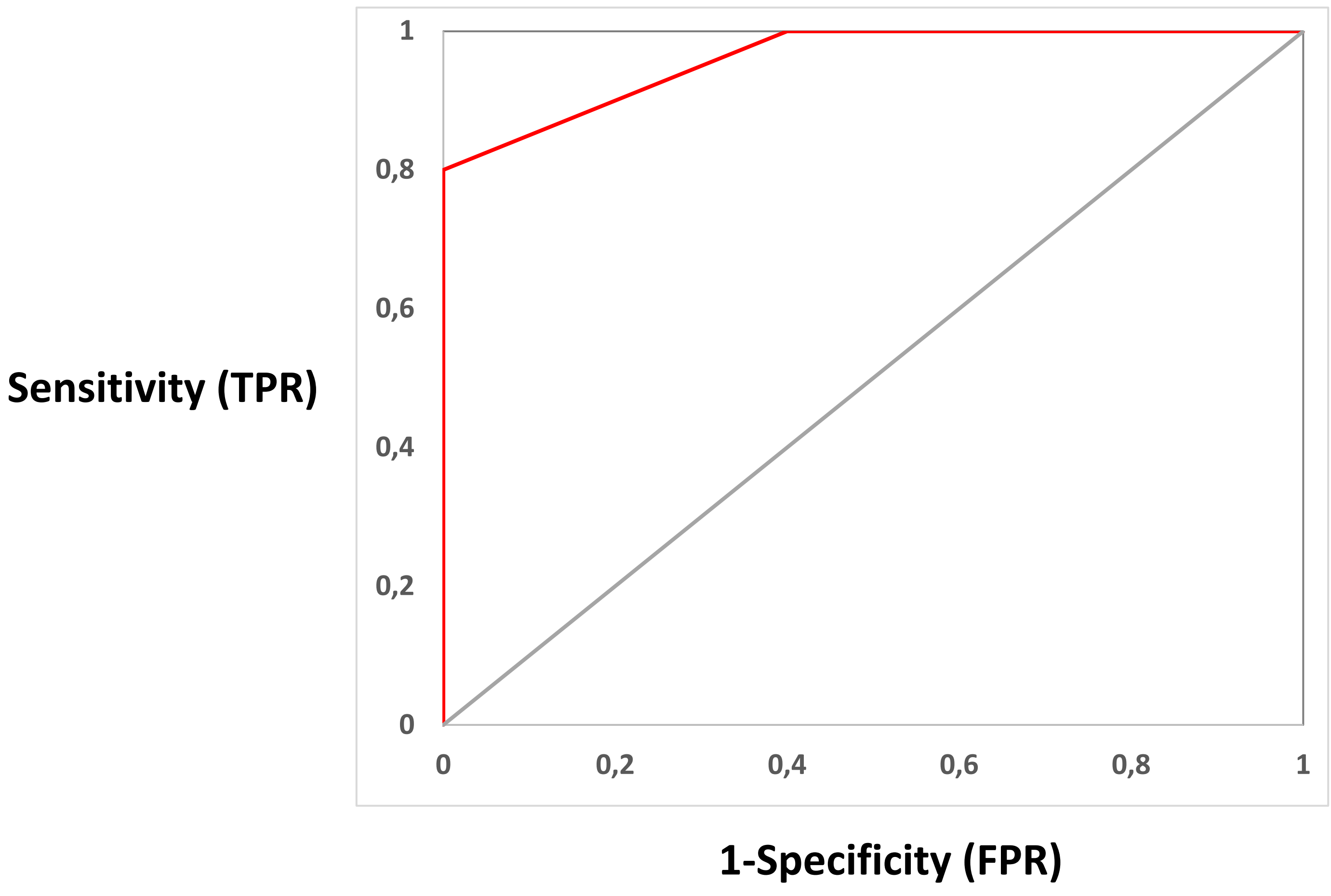

hsa-miR-199a-3p

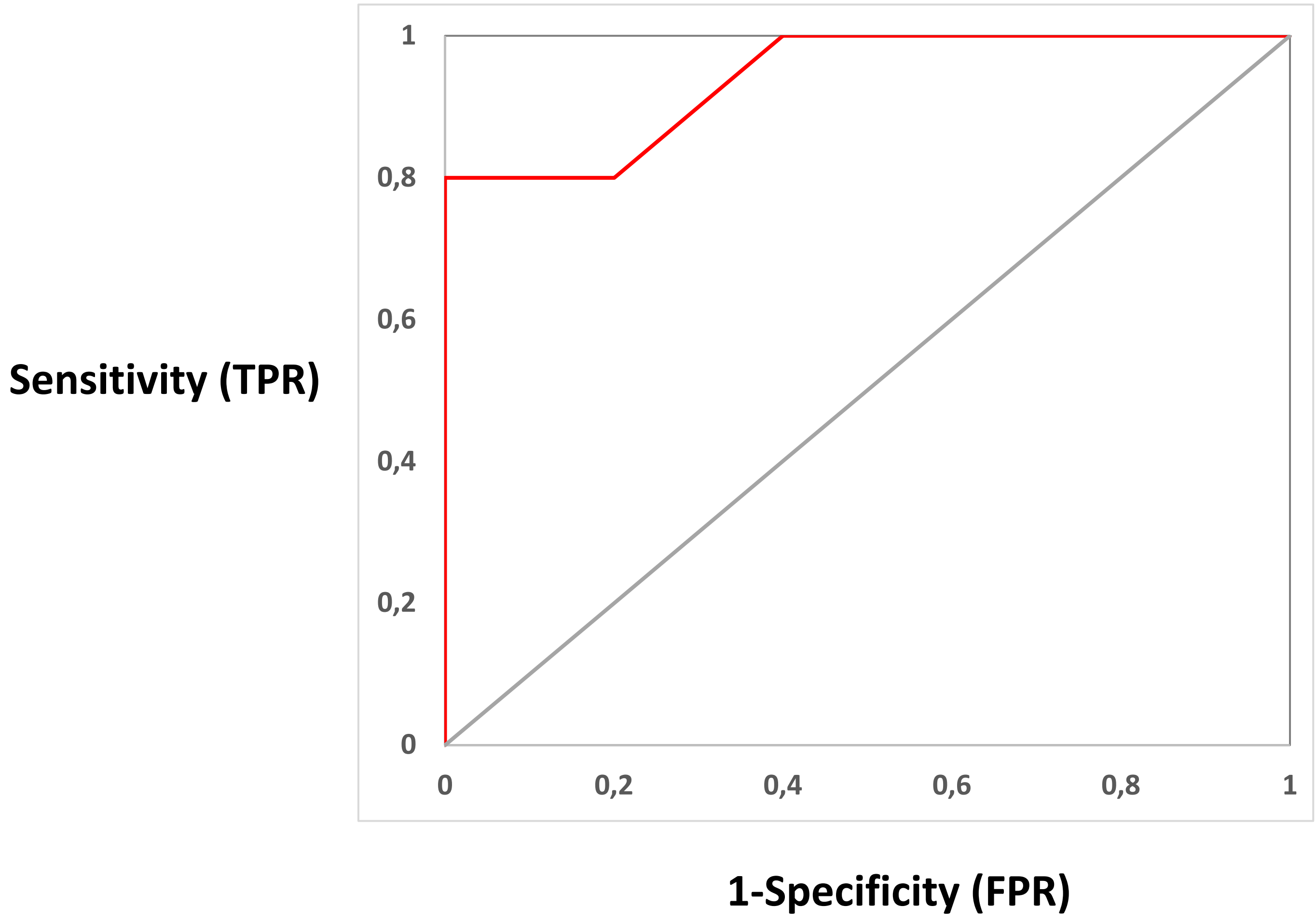

hsa-miR-199b-3p

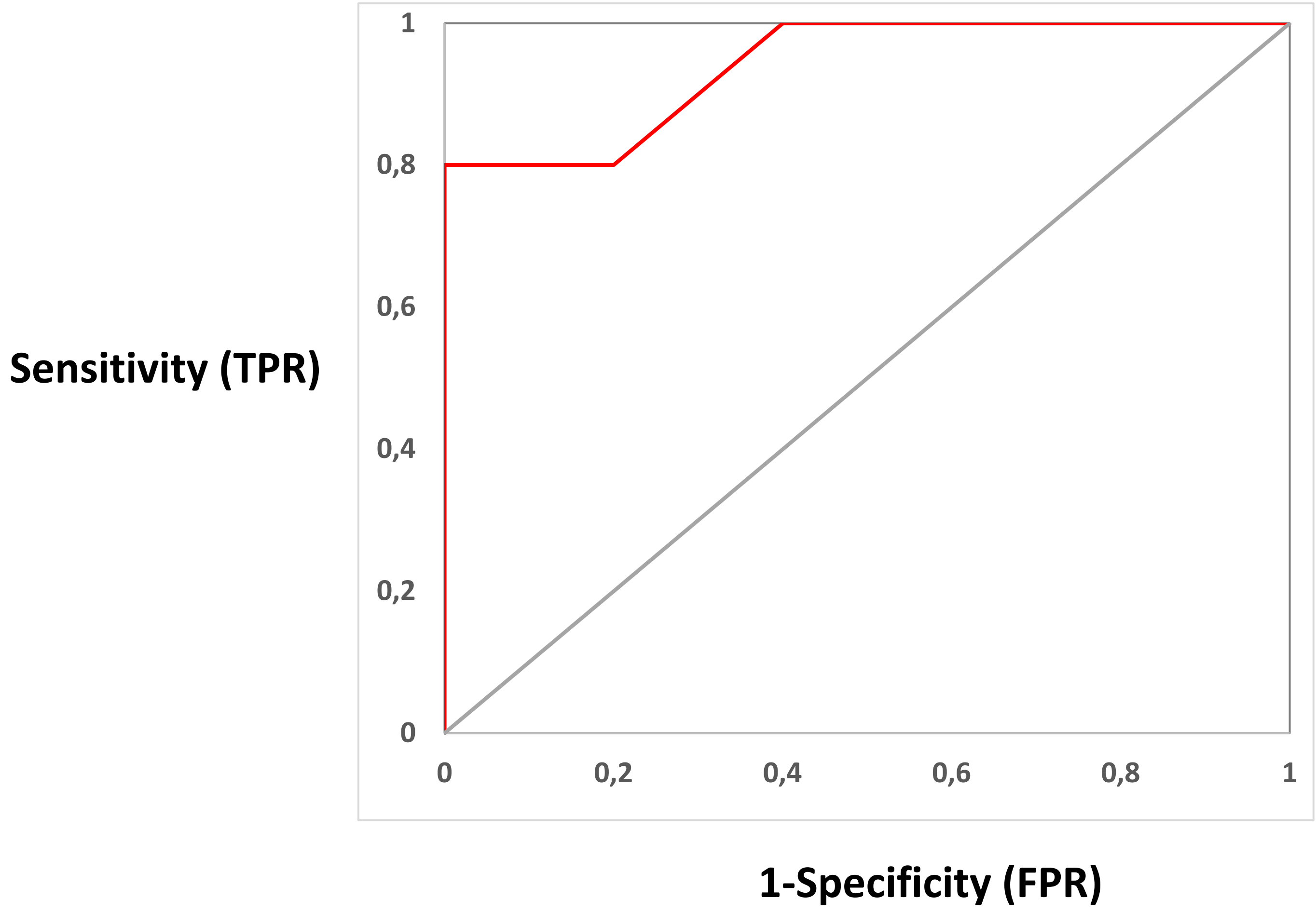

hsa-miR-520d-3p

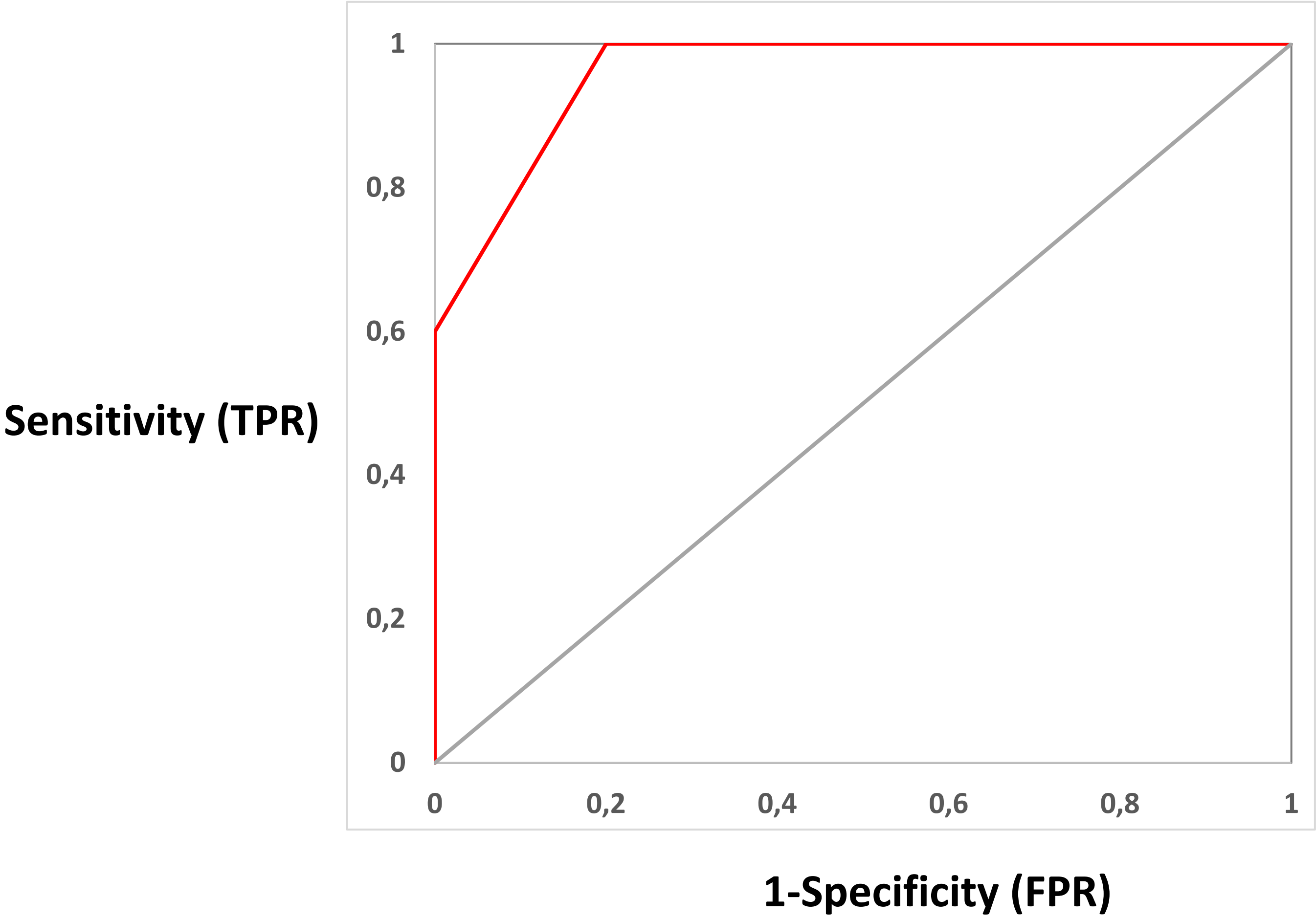

hsa-miR-548a-3p

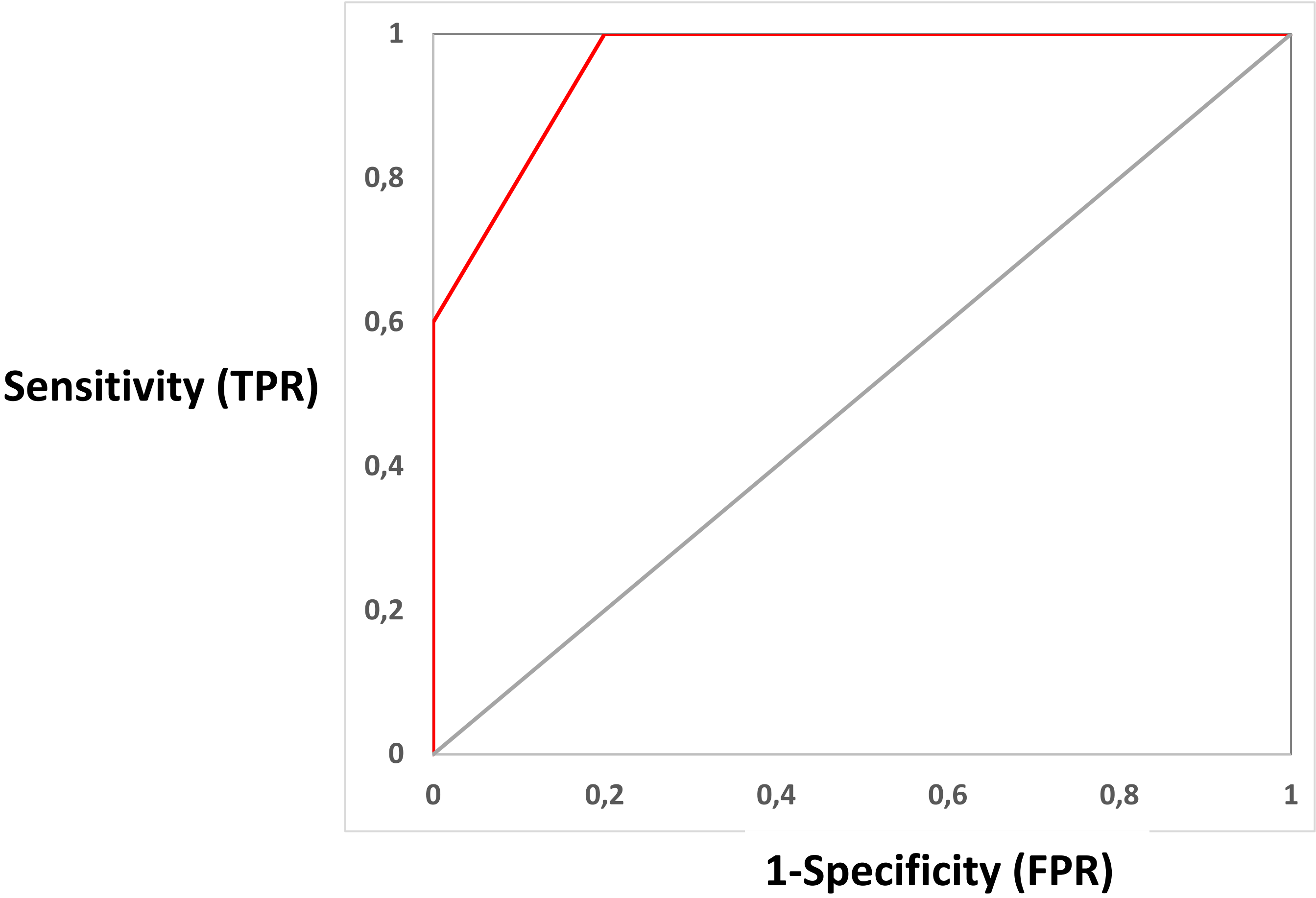

hsa-miR-656-3p

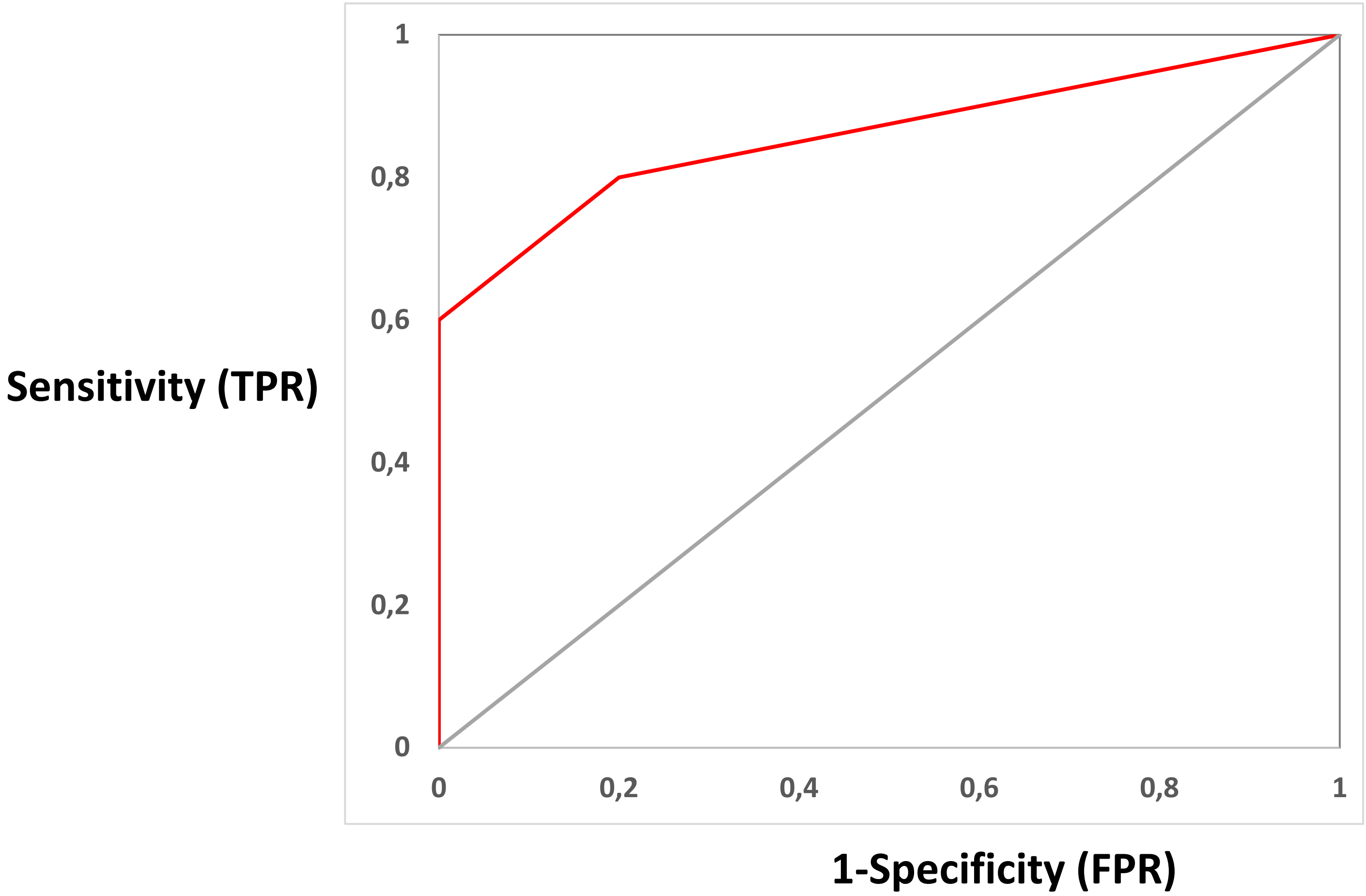

Supplement: Supplementary file 1 [file biomedicines-09-01470-s001.zip › biomedicines-1395414 suppplementary/Figure S1.pdf]
