# Supplementary material for: Network Analysis Integrating microRNA Expression Profiling with MRI Biomarkers and Clinical Data for Prostate Cancer Early Detection: A Proof of Concept Study
Source: Biomedicines. 2021 Oct 14;9(10):1470. doi: 10.3390/biomedicines9101470 (PMC8533640; doi:10.3390/biomedicines9101470)
Supplement: Supplementary file 1 [file biomedicines-09-01470-s001.zip › biomedicines-1395414 suppplementary/Supplementary File S1.pdf]

Supplementary Material

Additional results on the entire cohort of patients, including five patients with non-cancerous pathology, and four healthy donors (HD, age range 55-62).

The ANOVA test reported statistically significant differences (p-value $<10^{-4}$) between all possible pairs of groups, for liquid biopsy as well as prostate tissue (Figure 1a). These findings were confirmed by hierarchical clustering analysis (Figure 1b), where liquid biopsy data (EV and TP) form a well-separated group from the cluster created by tissue biopsy data (T), at the same time keeping healthy donors distinct from patients with benign and PCa patients.

In the whole cohort, the heatmaps of the module-trait association, corresponding to both EV and TP, showed a prevalence of negative correlations between the MEs of each module and the external traits (Figure 2a-b), indicating that miRNA expression levels tend to decrease in the disease status (Figure 3a-b) and with increasing values of the external traits (Figure 3c-f). The strongest correlations with disease status were found in the brown module from EV data and the blue module from TP data, leading to the identification of a common miRNAs signature. In fact, the two modules shared a 70% miRNA expression homology. Yet, in both modules, significant associations were found with ADC values of lesions (r=-0.41, p=0.07; r=-0.51, p=0.02) and with nADC (r=-0.34, p=0.1; r=-0.64, p= 0.002). By stratifying patients by median ADC (≤0.41 mm^2/s and >0.41 mm^2/s) and median nADC (≤0.62mm^2/s and >0.62 mm^2/s), we found that ADC and nADC values higher than the median corresponds to lower levels of miRNA expression (Figure 3c-f). Searching for miRNA drivers of the disease status, we ordered miRNAs of both modules (brown of EV and blue of TP) according to their module membership (MM) and gene significance (GS). Then, by setting a threshold on the values of these parameters, we selected miRNAs satisfying the conditions MM > 0.7 and a GS < -0.7 in both modules. Five common drivers of disease status were identified for further analysis: hsa-miR-19a-3p, hsa-miR-19b-3p, hsa-miR-126-3p, hsa-miR-144-3p, hsa-miR-185-5p. At the ROC analysis these five miRNA drivers for disease status reached an AUC > 0.98. Displayed in Figure 4 the miRNA-target gene interaction map constructed from these miRNAs of significance, and the functional enrichment analysis. Among the most enriched KEGG pathways “MicroRNAs in cancer” and cancer-related pathways such as “FoxO signaling pathway” and “MAPK signaling pathway”.

Figures

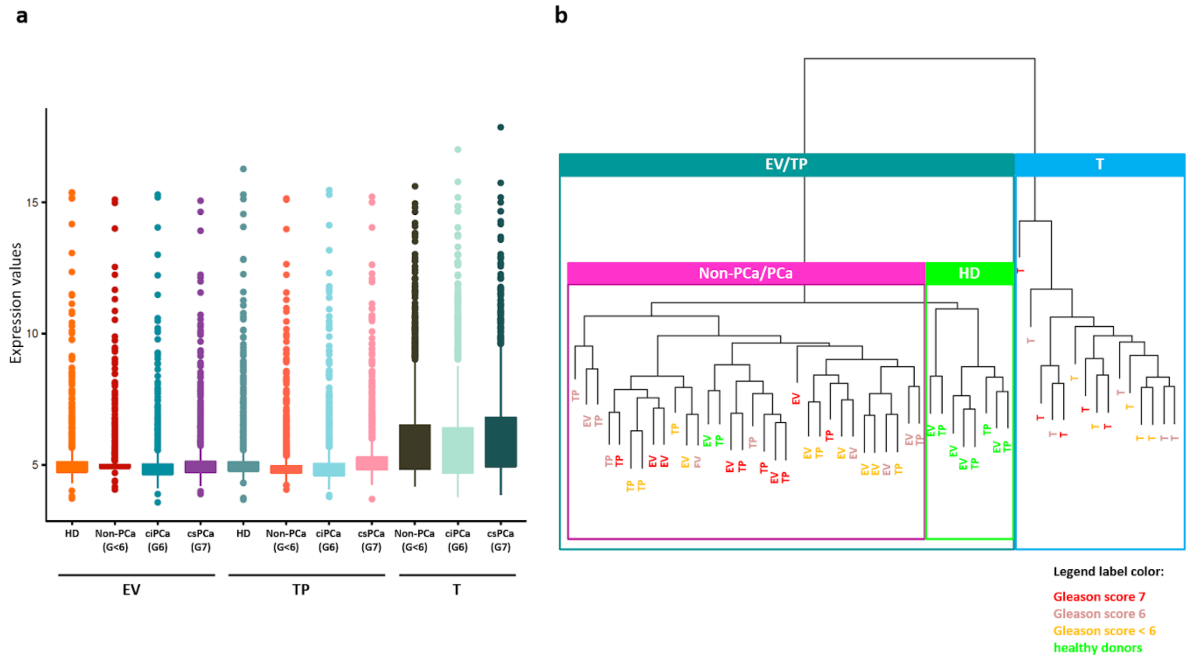

Figure 1. Exploration of miRNA expression profiles. a) Comparisons of miRNA expression values across both different patient status and different extraction sites. b) Hierarchical clustering analysis on liquid (EV and TP) and solid (T) biopsy data. HD, healthy donors; Non-PCa, non-prostate cancer; csPCa, clinically significant prostate cancer; ciPCa, clinically insignificant prostate cancer; G, Gleason score; EV, extracellular vesicles; TP, total plasma; T, tissue.

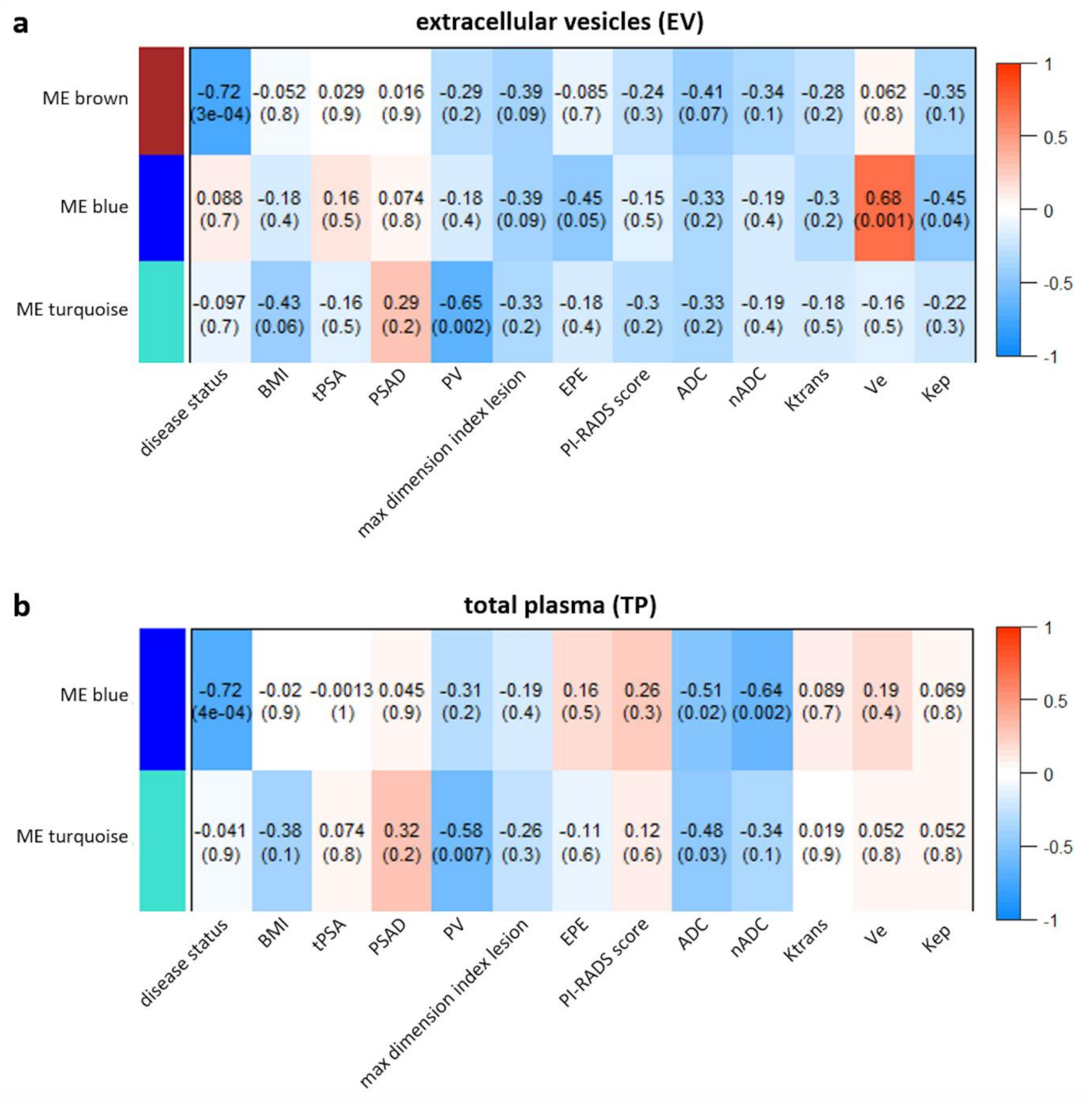

Figure 2. Weighted Gene Co-expression Network Analysis (WGCNA) analysis on EV and TP data of the whole cohort. *Module-trait associations.* Heatmap of the module-trait associations obtained by applying WGCNA on EV (a) and TP (b) data. In the heatmaps, each row corresponds to a module eigengene and each column to a trait of interest. Each cell contains the corresponding correlation and p-value. The heatmaps are color-coded by correlation according to the colour legend. EV, Extracellular Vesicles; TP, Total Plasma; ME, Module Eigengene; BMI, Body Mass Index; tPSA, total Prostate Specific Antigen; PSAD, Prostate Specific Antigen Density; EPE, Extra-Prostatic Extension; ADC, Apparent Diffusion Coefficient; nADC, normalized Apparent Diffusion Coefficient.

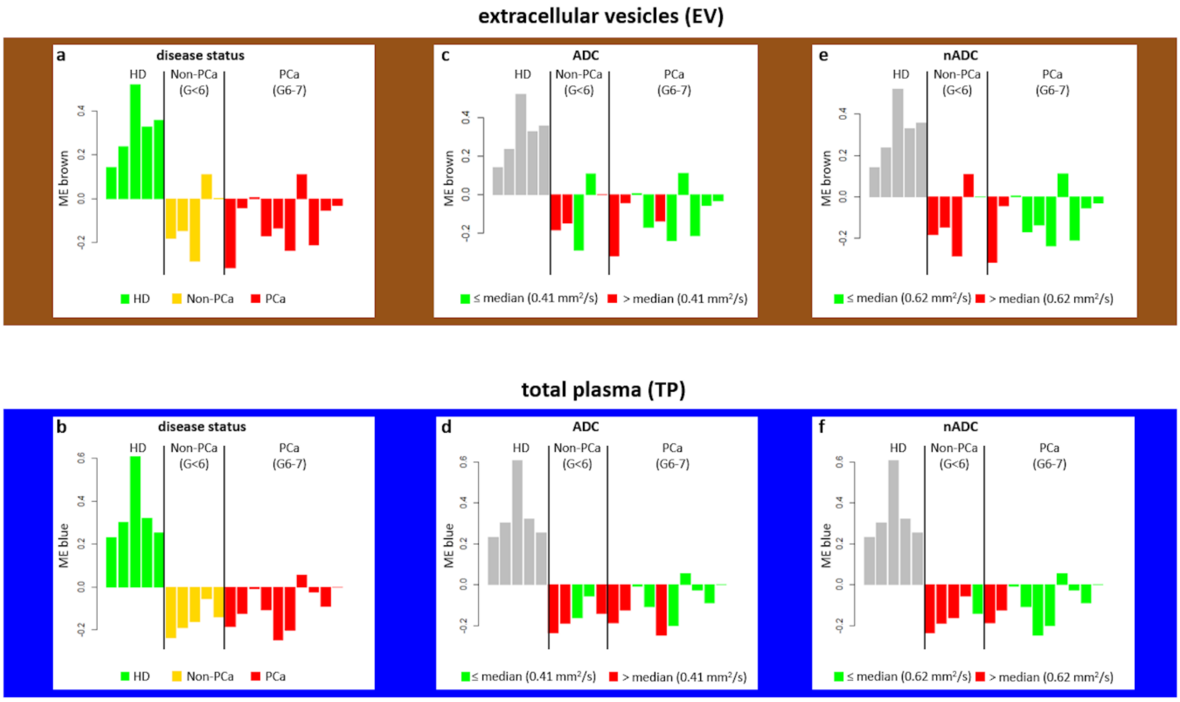

Figure 3. Module-trait association. Bar plots of the expression levels (y-axis) of brown module eigengene for EV data (a-c-e) and of blue module eigengene for TP data (b-d-f), across healthy, Non-PCa, and PCa samples (x-axis). Expression levels of the module eigengenes were log2-transformed and z-score normalized. Grey colour was used to indicate not available data. EV, Extracellular Vesicles; TP, Total Plasma; PCa, Prostate Cancer; ADC, Apparent Diffusion Coefficient; nADC, normalized Apparent Diffusion Coefficient

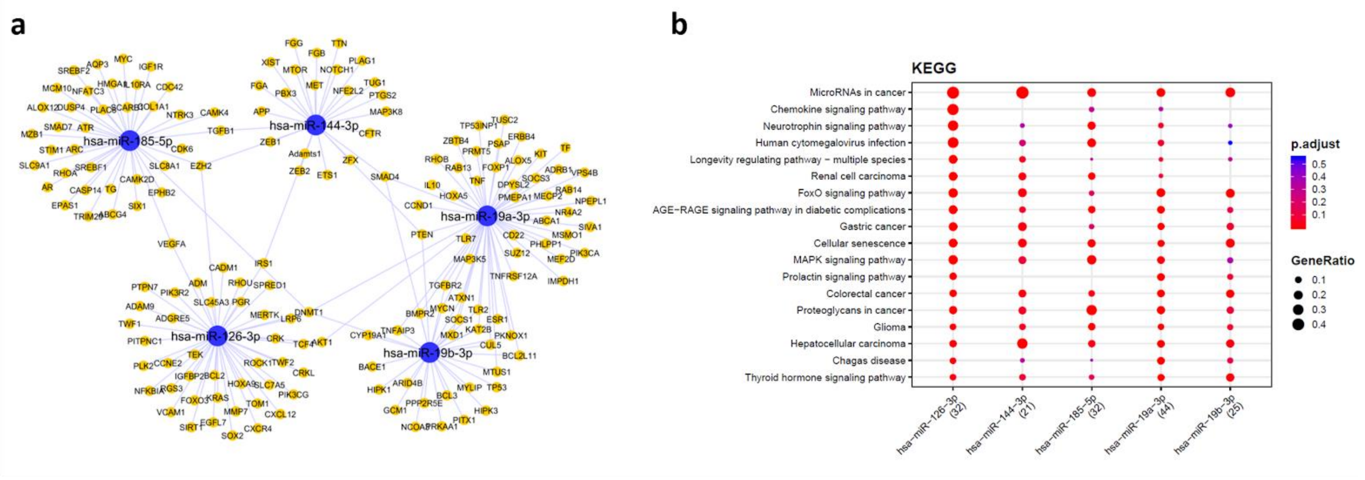

Figure 4. miRNA drivers. a) *miRNA-target interaction network for miRNA drivers shared between TP blue module and EV brown module*. The network shows the experimentally validated miRNA-target interactions retrieved from MIENTURNET. Blue dots represent miRNAs, yellow dots represent miRNA targets. b) *KEGG pathways enrichment analysis.* The main enrichment results for the targets of the miRNAs appearing in the network are presented as a dot plot, where the Y-axis reports the annotation categories (i.e., KEGG pathways) and the X-axis reports the miRNAs with the number of recognized targets (i.e., number of targets with at least one annotation) in round brackets. The colours of the dots represent the adjusted p-values, whereas the size of the dots represents gene ratio (i.e., the number of miRNA targets found annotated in each category over the total number of recognized targets indicated in round brackets). EV, Extracellular Vesicles; TP, Total Plasma; KEGG, Kyoto Encyclopaedia of Genes and Genomes
